# Reconstruction with megaprosthesis for recurrent nonunion of an atypical periprosthetic femoral fracture in an elderly patient

**DOI:** 10.1093/jscr/rjag768

**Published:** 2026-09-01

**Authors:** Yuto Nishiura, Tomohiro Yoshizawa, Tomofumi Nishino, Toshiki Muramatsu, Yohei Yanagisawa, Ryunosuke Watanabe, Yasuhiro Homma

**Affiliations:** Department of Orthopaedic Surgery, Institute of Medicine, University of Tsukuba, 1-1-1, Tennodai, Tsukuba, Ibaraki 305-8575, Japan; Department of Orthopaedic Surgery, University of Tsukuba Hospital, 2-1-1, Amakubo, Tsukuba, Ibaraki 305-8576, Japan; Department of Orthopaedic Surgery, Tsukuba Medical Center Hospital, 1-3-1, Amakubo, Tsukuba, Ibaraki 305-8558, Japan; Department of Orthopaedic Surgery, Institute of Medicine, University of Tsukuba, 1-1-1, Tennodai, Tsukuba, Ibaraki 305-8575, Japan; Department of Orthopaedic Surgery, University of Tsukuba Hospital, 2-1-1, Amakubo, Tsukuba, Ibaraki 305-8576, Japan; Department of Orthopaedic Surgery, Institute of Medicine, University of Tsukuba, 1-1-1, Tennodai, Tsukuba, Ibaraki 305-8575, Japan; Department of Orthopaedic Surgery, Tsukuba Gakuen Hospital, 2573-1, Kamiyokoba, Tsukuba, Ibaraki 305-0854, Japan; Department of Emergency and Critical Care Medicine, Institute of Medicine, University of Tsukuba, 1-1-1, Tennodai, Tsukuba, Ibaraki 305-8575, Japan; Department of Orthopaedic Surgery, Institute of Medicine, University of Tsukuba, 1-1-1, Tennodai, Tsukuba, Ibaraki 305-8575, Japan; Department of Orthopaedic Surgery, Institute of Medicine, University of Tsukuba, 1-1-1, Tennodai, Tsukuba, Ibaraki 305-8575, Japan

**Keywords:** periprosthetic femoral fracture, atypical femoral fracture, megaprosthesis, nonunion, revision arthroplasty

## Abstract

An 89-year-old woman who had previously undergone cementless hemiarthroplasty for a left femoral neck fracture sustained a Vancouver type C periprosthetic femoral fracture with radiographic features suggestive of an atypical femoral fracture. Both attempts at internal fixation resulted in nonunion accompanied by plate failure. Given the well-fixed stem and the anticipated difficulty of stem extraction owing to severe proximal femoral cortical thinning, we elected to perform megaprosthetic reconstruction. Immediate full weight-bearing was permitted postoperatively, and the patient was discharged home seven weeks after surgery. In elderly patients in whom osseous union is improbable and preservation of functional independence through early weight-bearing is paramount, megaprosthetic reconstruction represents a viable salvage option.

## Introduction

Periprosthetic femoral fracture (PFF) is increasing in incidence in parallel with the aging population and the growing volume of arthroplasty procedures, and is associated with poor functional outcomes and elevated mortality [1]. Elderly patients are particularly frequently unable to regain their preinjury level of activities of daily living (ADL). Prolonged recumbency not only predisposes to systemic complications, such as hypostatic pneumonia and venous thromboembolism, but also leads to irreversible functional deterioration secondary to disuse syndrome. Therefore, early mobilization to preserve functional independence constitutes a therapeutic objective of equal importance to osseous union. Previous reports have demonstrated comparable clinical outcomes between internal fixation and revision arthroplasty, underscoring the need for individualized decision-making informed by each patient’s overall clinical status [2].

## Case report

An 89-year-old woman (height 133 cm, weight 36 kg, and body mass index 20 kg/m^2^) was referred to our institution following a low-energy fall. Her past medical history was notable for cementless hemiarthroplasty for a left femoral neck fracture performed at another institution 27 years prior, and intramedullary nailing for a right femoral shaft fracture 20 years previously. She had a history of hypertension and osteoporosis and was receiving nifedipine, imidapril, calcitriol, and alendronate, the latter for more than 20 years. She had remained independently ambulatory prior to the injury.

**Figure 1:**
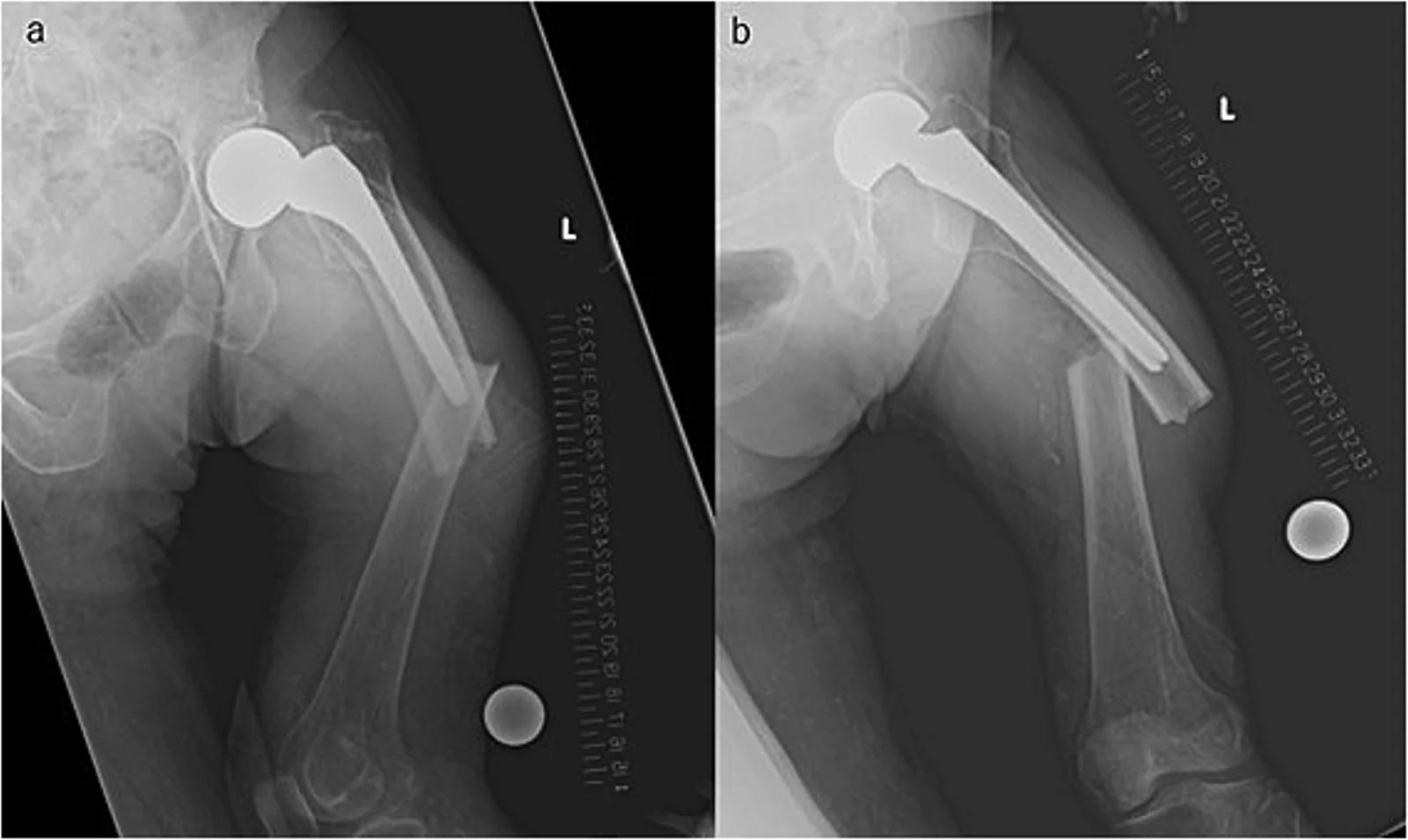
Anteroposterior (a) and lateral (b) plain radiographs obtained at presentation. A transverse, non-comminuted fracture with lateral cortical thickening and femoral bowing is demonstrated, consistent with the radiographic appearance of an atypical femoral fracture.

**Figure 2:**
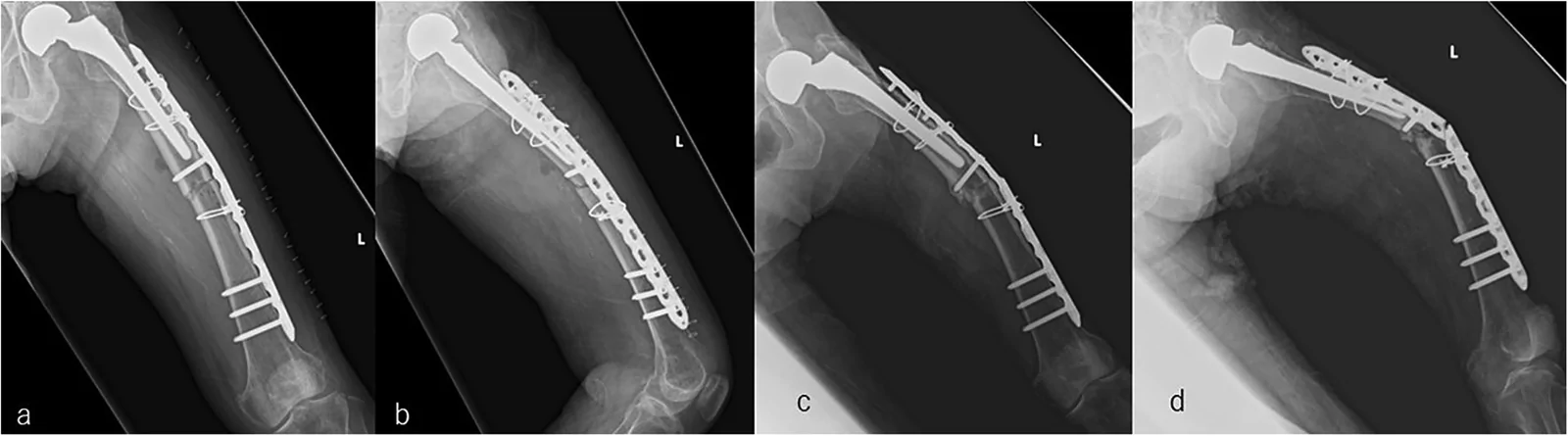
Internal fixation was performed with a plate contoured to accommodate the lateral femoral bow (a and b). Plate failure occurred at seven months postoperatively (c and d).

**Figure 3:**
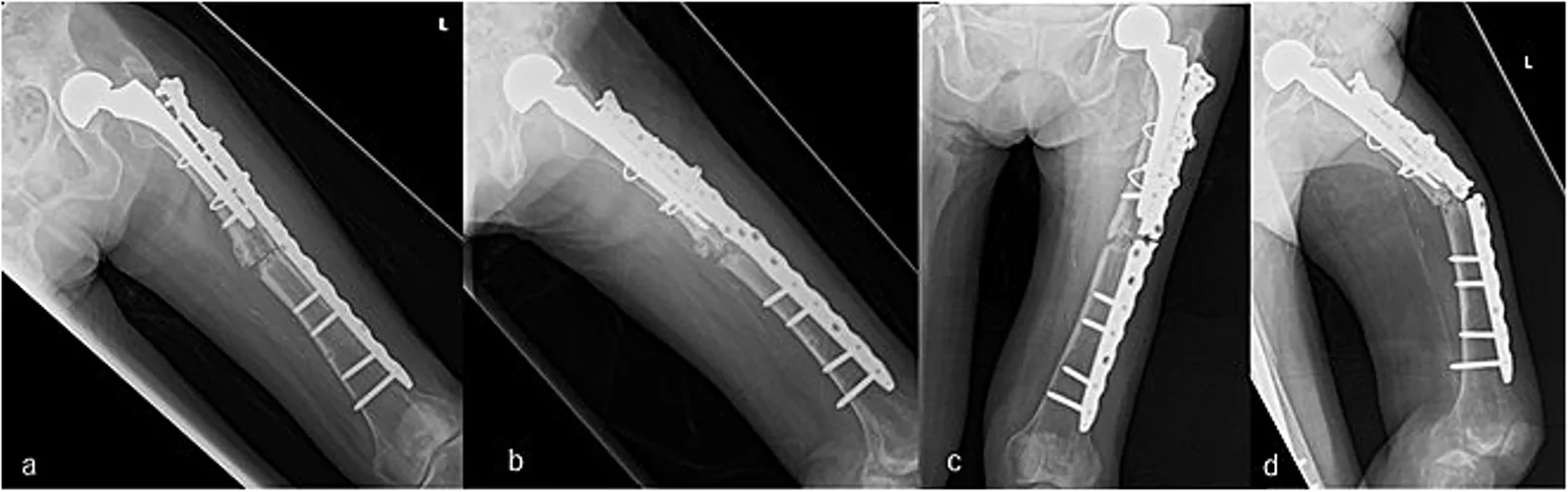
Internal fixation was performed in combination with a valgus corrective femoral osteotomy to realign the femur with the plate (a and b). Plate failure recurred at 22 months postoperatively (c and d).

**Figure 4:**
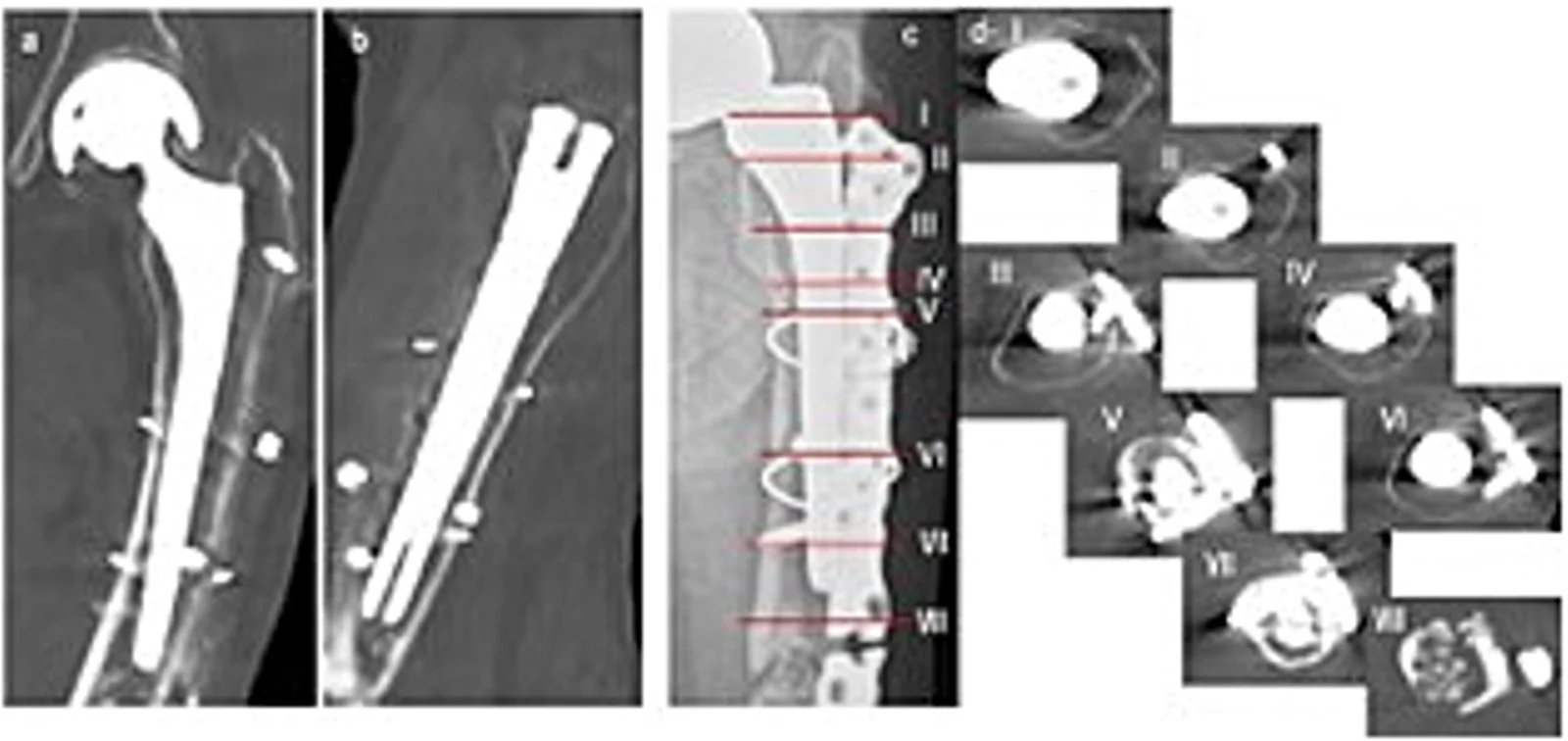
Plain CT imaging. (a) Coronal reconstruction, (b) sagittal reconstruction, (c) scout view, and (d) axial CT images at the levels indicated in (c) (d-I to d-VIII). These images demonstrate no circumferential gap between the stem and femur from proximal to distal. Marked cortical thinning of the proximal femur attributable to stress shielding is also evident.

Plain radiographs revealed a left PFF, classified as Vancouver type C [3] (Fig. 1). The fracture demonstrated a transverse, non-comminuted pattern with lateral cortical thickening and femoral bowing, findings concordant with the radiographic appearance of an atypical femoral fracture (AFF). Although periprosthetic location constitutes an exclusion criterion in the formal diagnostic criteria for AFF, the fracture fulfilled all major criteria [4]. As the initial surgical strategy, internal fixation was performed using a broad locking compression plate (LCP; DePuy Synthes, Oberdorf, Switzerland) contoured to accommodate the lateral femoral bow; however, plate failure occurred at seven months postoperatively (Fig. 2). The revision procedure combined a valgus corrective femoral osteotomy with internal fixation using a non-contact bridging plate (NCB plate; Zimmer Biomet, Warsaw, IN, USA), and teriparatide was administered postoperatively. Despite these measures, plate failure recurred 22 months after the second fixation (Fig. 3). Plain radiographs and computed tomography (CT) imaging demonstrated no periprosthetic radiolucent lines, with spot welds at the coating border confirming a well-fixed stem. Marked cortical thinning of the proximal femur attributable to stress shielding was also noted (Fig. 4).

Given the patient’s advanced age, relatively preserved functional status, two prior failures of internal fixation, anticipated poor bone healing owing to AFF-like pathology, the presence of a well-fixed stem, and the anticipated technical difficulty of stem extraction in the setting of severe proximal femoral cortical thinning, reconstruction with a megaprosthesis was selected.

**Figure 5:**
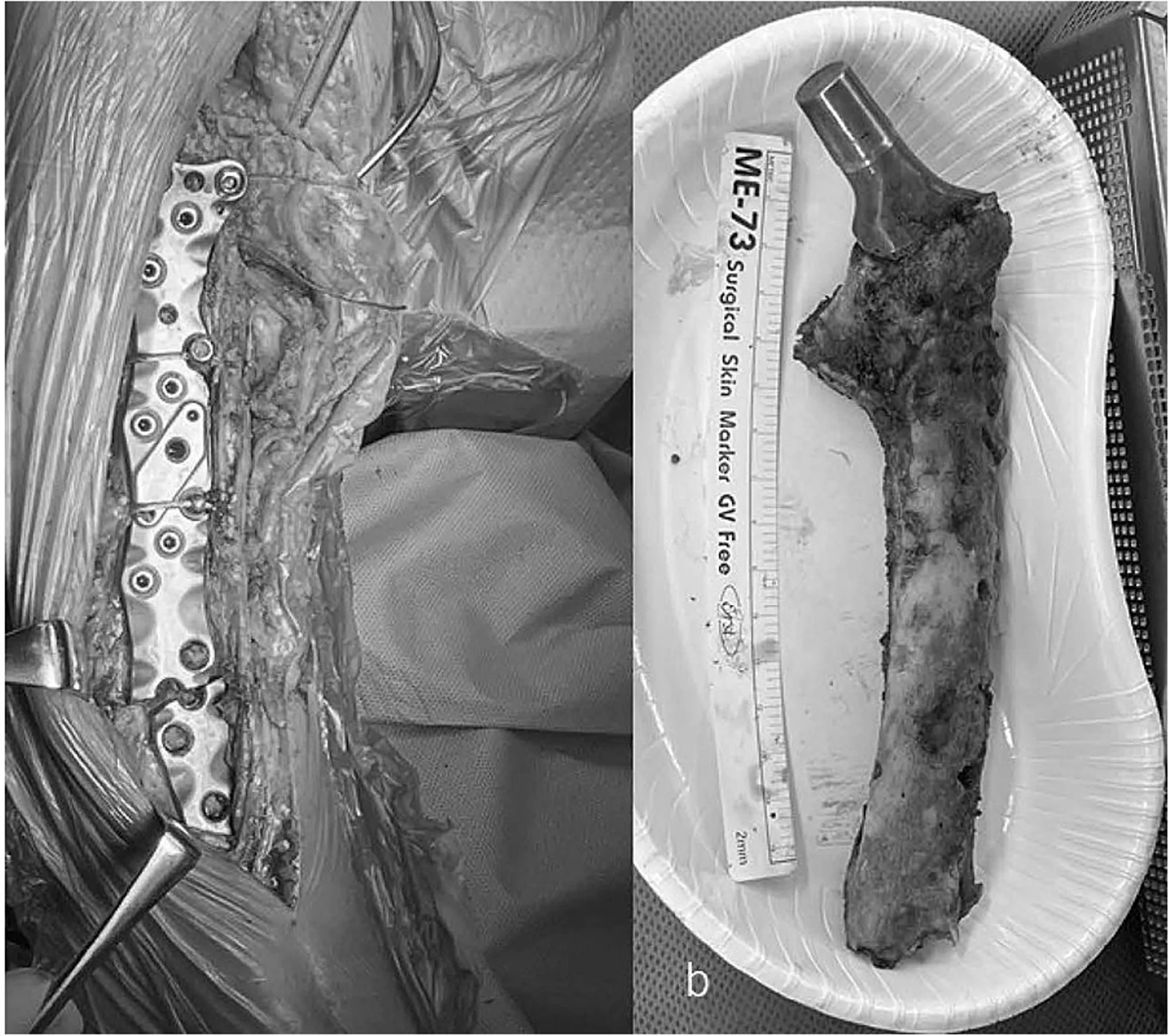
Through a posterior approach, the failed plate was removed (a). Because the stem was firmly fixed to the femur, a greater trochanteric osteotomy was performed and the stem was extracted en bloc with the proximal bone fragment (b).

**Figure 6:**
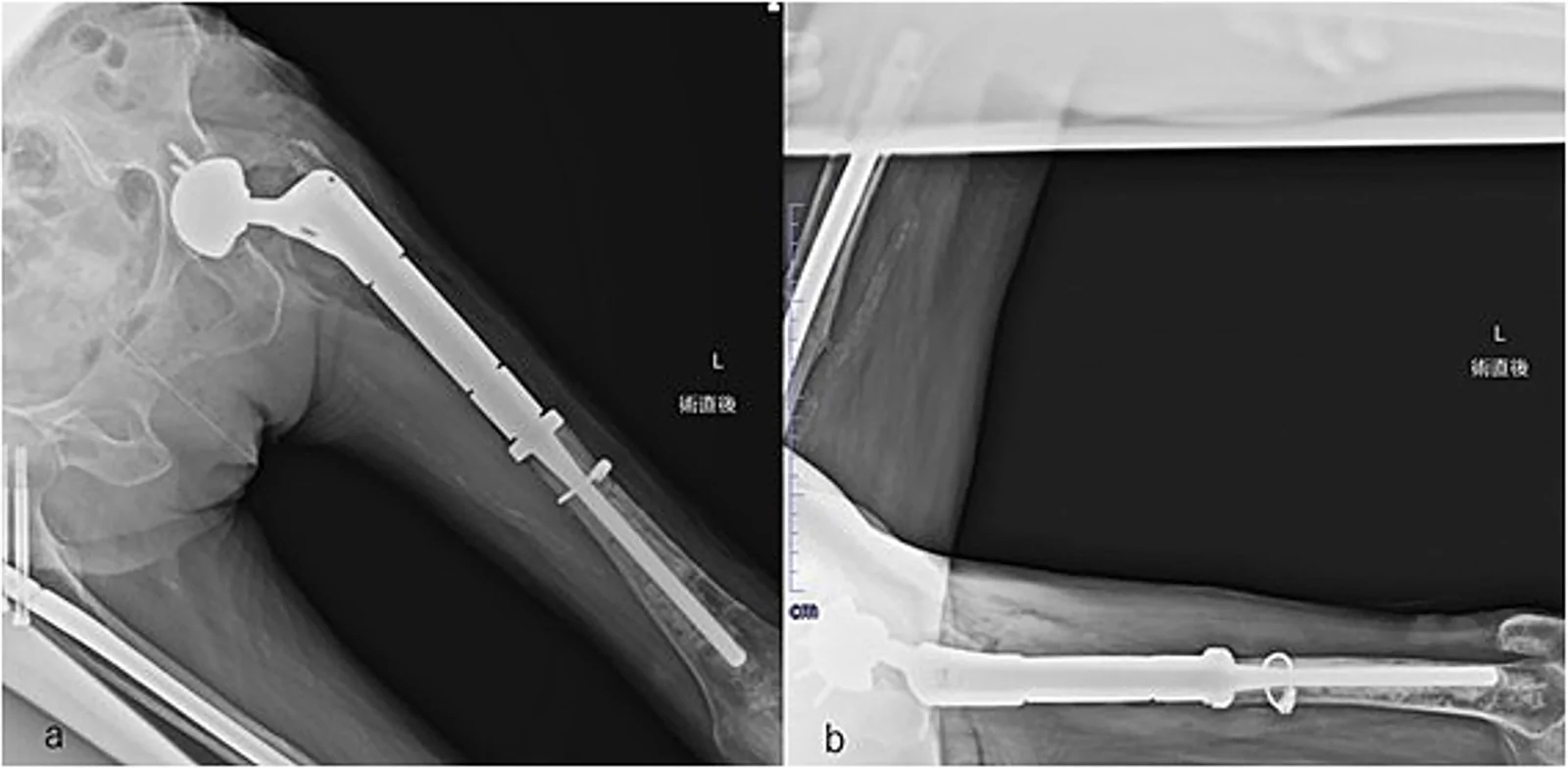
A G7 acetabular component (Zimmer Biomet, Warsaw, IN, USA) with a dual mobility articulation was implanted. The femur was osteotomized distal to the nonunion site, and reconstruction was performed using an Orthopedic Salvage System (Zimmer Biomet, Warsaw, IN, USA) with cement fixation of the stem (a and b).

The hip was approached posteriorly, and the retained plate was removed. Because the stem was firmly fixed to the femur, a greater trochanteric osteotomy was performed, and the stem was extracted en bloc with the proximal bone fragment (Fig. 5). A G7 acetabular component (Zimmer Biomet, Warsaw, IN, USA) incorporating a dual mobility articulation was implanted on the acetabular side. The femur was osteotomized distal to the nonunion site, and reconstruction was performed using an Orthopedic Salvage System (Zimmer Biomet, Warsaw, IN, USA) with cement fixation of the stem (Fig. 6). The operative time was 4 h and 20 min and the intraoperative blood loss was 130 ml.

**Figure 7:**
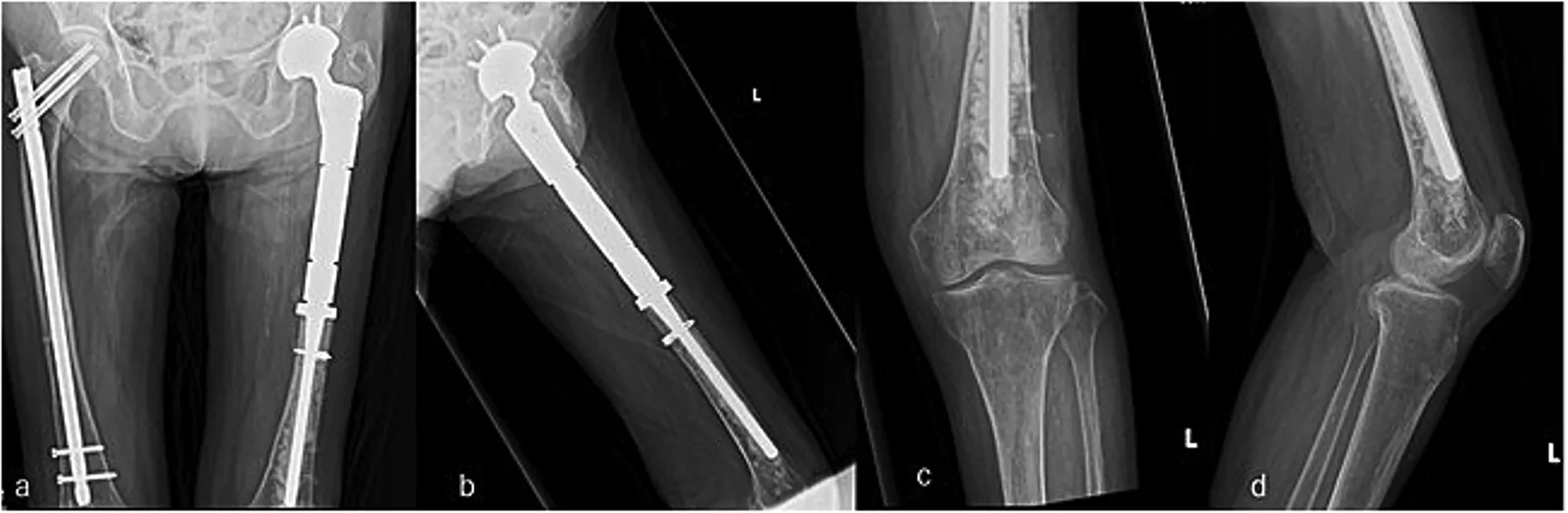
Plain radiographs at six months postoperatively (a–d). No evidence of implant loosening or new fracture is identified.

Immediate postoperative full weight-bearing was permitted, and the patient was ambulating with a walker by postoperative Week 2. Following inpatient rehabilitation, she was discharged home seven weeks postoperatively. By three and a half months, she had achieved outdoor ambulation with a single cane. At six months postoperatively, her ADL were maintained, and radiographic evaluation revealed no signs of implant loosening or new fractures (Fig. 7).

## Discussion

Megaprosthetic reconstruction successfully salvaged a recurrent nonunion of an atypical PFF after two failed fixation procedures. In elderly patients, this strategy permits immediate weight-bearing and may preserve functional independence.

Advanced age, osteoporosis, and prior arthroplasty are all well-established risk factors for PFF [1]. The present patient harbored each of these factors, indicating a high preoperative risk for PFF.

For Vancouver type C fractures with a stable stem, osteosynthesis is generally the treatment of choice [4]. However, in cases exhibiting AFF-like features, outcomes are inconsistent, with delayed union and nonunion reported at higher rates [4, 5].

The concept of “peri-implant atypical femoral fracture” has recently been proposed to describe AFF-like fractures occurring around joint prostheses or internal fixation devices [6]. These fractures are characterized by lateral cortical thickening, a transverse non-comminuted pattern, and a low-energy mechanism, with stress concentration at the implant interface implicated in their pathogenesis [6]. The present case fulfilled all of these features, supporting that AFF-like pathology contributed to the repeated plate failures. In retrospect, double plating might have constituted a more biomechanically robust strategy. Double plate fixation has been reported to improve union rates and reduce reoperation rates relative to single plate fixation, and represents a reliable technique for achieving a stable construct [7]. However, the attendant increase in surgical invasiveness limits its applicability to all patients.

Following two episodes of plate failure, the available salvage options comprised repeat internal fixation, long-stem revision arthroplasty, and megaprosthesis reconstruction. Repeat internal fixation preserves bone stock but remains contingent on bone healing, which is unfavorable in AFF-like pathology. Were this approach undertaken, double plating [7] combined with bone grafting or other adjuncts would be required. Even if union were achieved, the prolonged period of restricted weight-bearing required in the interim would be expected to result in considerable functional decline in an elderly patient. Revision arthroplasty offers load-bypassing fixation; however, its application was limited by severe cortical thinning of the proximal femur and the presence of a well-fixed stem. Intraoperative findings confirmed that stem extraction would have been technically considerably difficult in this case.

In contrast, megaprosthesis reconstruction does not require bone healing and permits immediate full weight-bearing, potentially minimizing disuse-related deterioration and contributing to improved functional outcomes in the elderly population. Calori *et al.* reported favorable outcomes with megaprosthesis in non-oncologic indications [8], and Lundh *et al.* reported high rates of ambulation recovery and return to independent living in patients with PFF [9].

However, megaprosthesis is associated with substantial complications, including dislocation and infection; hip dislocation rates of 6%–42% have been reported [7]. In the present case, a dual mobility cup was used to reduce dislocation risk and facilitate safe early rehabilitation [10].

In summary, for elderly patients with AFF-like PFF or recurrent fixation failure in whom bone healing is improbable, a strategy prioritizing early weight-bearing to avoid functional decline appears preferable to persistence with osteosynthesis in anticipation of union. In such cases, megaprosthesis reconstruction represents a compelling salvage option.

## Conclusion

We report a case of recurrent nonunion of an atypical PFF successfully managed by megaprosthetic reconstruction, with satisfactory functional recovery. This procedure represents a viable surgical option when bone healing is unlikely or early weight-bearing is essential for maintaining functional independence in elderly patients.

## References

[1] Lindahl H, Malchau H, Oden A et al. Risk factors for failure after treatment of a periprosthetic femoral fracture. J Bone Joint Surg Br 2006;88:26–30. 10.1302/0301-620X.88B1.16314 16365115

[2] Marino S, Vitiello R, Cauteruccio M et al. Treatment options for proximal periprosthetic femoral fractures in total hip arthroplasty: a single center experience. Eur Rev Med Pharmacol Sci 2022;26:113–8. 10.26355/eurrev_202211_30290 36448863

[3] Duncan CP, Masri BA. Fractures of the femur after hip replacement. Instr Course Lect 1995;44:293–304. 7797866

[4] Shane E, Burr D, Abrahamsen B et al. Atypical subtrochanteric and diaphyseal femoral fractures: second report of a task force of the American Society for Bone and Mineral Research. J Bone Miner Res 2014;29:1–23. 10.1002/jbmr.1998 23712442

[5] Egol KA, Park JH, Rosenberg ZS et al. Healing delayed but generally reliable after bisphosphonate-associated complete femur fractures. J Bone Joint Surg Am 2014;96:284–90. 10.2106/JBJS.L.01717 PMC4117878 23604648

[6] Lee JYY, Soh T, Howe TS et al. Periprosthetic atypical femoral fractures associated with bisphosphonate use. Acta Orthop 2015;86:639–43. 10.3109/17453674.2015.104383126446801

[7] Lodde MF, Katthagen JC, Schopper CO et al. Union rates and functional outcome of double plating of the femur: systematic review of the literature. Arch Orthop Trauma Surg 2021;142:1009–30. 10.1007/s00402-021-03767-6 33484313 PMC9110521

[8] Calori GM, Colombo M, Malagoli E et al. Megaprosthesis in non-oncologic patients. Injury 2014;45:S116–20. 10.1016/j.injury.2014.10.034 25457330

[9] Lundh F, Sayed-Noor AS, Brosjö O et al. Megaprosthesis reconstruction for complex femoral fractures. Eur J Orthop Surg Traumatol 2014;24:125–30. 10.1007/s00590-013-1177-7 23689913

[10] Philippot R, Adam P, Reckhaus M et al. Prevention of dislocation in total hip revision surgery using a dual mobility design. Clin Orthop Relat Res 2009;467:389–95. 10.1007/s11999-008-0596-9 19656750

